# Evaluating Allied Health Clinical Placement Performance: Protocol for a Modified Delphi Study

**DOI:** 10.2196/44020

**Published:** 2023-08-31

**Authors:** Lisa Simmons, Ruth Barker, Fiona Barnett

**Affiliations:** 1 James Cook University Townsville, Queensland Australia

**Keywords:** clinical placement, cost of placement, learning outcomes, student experience, student-led health care services

## Abstract

**Background:**

University-affiliated student-led health care services have emerged in response to the challenges faced by universities in securing quality clinical placements for health care students. Evidence of the health care benefits and challenges of student-led health care services is growing, while evidence of clinical placement performance remains variable and not generalizable. Though there have been previous attempts to develop a framework for evaluation of clinical placement performance, concerns have been raised about the applicability of these frameworks across the various placement settings. Additionally, the perspectives of all key stakeholders on the critical areas of clinical placement performance have yet to be considered.

**Objective:**

This study’s objective is to gather information on areas of measurement related to student learning outcomes, experience of placement, and costs of placement and then develop consensus on which of those areas need to be included in a framework for evaluation of clinical placement performance within the context of student-led health care services. The aim of this paper is to outline a protocol for a modified Delphi study designed to gain consensus on what is important to measure when evaluating an allied health clinical placement.

**Methods:**

We will recruit up to 30 experts to a heterogeneous expert panel in a modified Delphi study. Experts will consist of those with firsthand experience either coordinating, supervising, or undertaking clinical placement. Purposive sampling will be used to ensure maximum variation in expert panel member characteristics. Experts’ opinions will be sought on measuring student learning outcomes, student experience, and cost of clinical placement, and other areas of clinical placement performance that are considered important. Three rounds will be conducted to establish consensus on what is important to measure when evaluating clinical placement. Each round is anticipated to yield both quantitative data (eg, percentage of agreement) and qualitative data (eg, free-text responses). In each round, quantitative data will be analyzed descriptively and used to determine consensus, which will be defined as ≥70% agreement. Qualitative responses will be analyzed thematically and used to inform the subsequent round. Findings of each round will be presented, both consensus data and qualitative responses in each subsequent round, to inform expert panel members and to elicit further rankings on areas of measurement yet to achieve consensus.

**Results:**

Data analysis is currently underway, with a planned publication in 2024.

**Conclusions:**

The modified Delphi approach, supported by existing research and its ability to gain consensus through multiround expert engagement, provides an appropriate methodology to inform the development of a framework for the evaluation of clinical placement performance in allied health service.

**International Registered Report Identifier (IRRID):**

DERR1-10.2196/44020

## Introduction

Universities face many challenges in sourcing and securing clinical placements for health students [1]. For regional universities, there is the added pressure of limited health care services from which to secure sufficient clinical placements [2]. The cost associated with paid placements is also a major concern, as paid placements can potentially create issues around placement outcomes and add to the financial burden on universities [3,4]. For example, the question for universities securing paid placements is whether the student learning outcomes or the student experiences achieved in these placements are equivalent to the amount paid. Conversely, the question may be whether an equivalent or greater outcome could be achieved through an alternate clinical placement. Placement providers also report increased costs associated with supervising students on clinical placement, including loss of productivity when working with inexperienced students, increased recruitment requirements to manage the additional workload, supervision of students on top of clinical workloads, and procuring and maintaining state-of-the-art equipment for teaching [5]. Cost of clinical placement to the clinical placement provider has been estimated to be an average of Aus $34,326 (US $22,531.3) per year for 1 student [5], influencing a health care provider’s ability to offer placements without compensation [4]. As a result, university-affiliated student-led health care services have emerged to address the many challenges faced by universities in securing quality clinical placements for health care students [6].

Student-led health care services are often seen as innovative health care models providing much-needed services to disadvantaged or underserviced populations [7]. There is mounting evidence supporting the benefits of student-led health care services as both adequate providers of care and attractive educational opportunities [8]. In a recent study, allied health students co-designed and implemented a health service in a remote Aboriginal community in northern Australia, where students learned how to adapt their skills and approaches to the cultural needs of the Aboriginal community [9]. However recent reviews of the outcomes of student-led health care services more broadly, indicate the need for more rigorous reporting [10], with more specific attention to students’ skill development, knowledge, and behavior required [11]. If students are not adequately prepared to operate in a fashion similar to that of qualified health care professionals, then it is possible that undertaking placement in a student-led health care service may be detrimental to their learning outcomes and overall experience of placement. Conversely, providing students with the ability to operate in authentic clinical environments where they can collaborate and regulate their own learning needs may improve overall student experience. Current findings from evaluations of student-led health care services as providers of clinical placement lack generalizability [11,12] and there is little consensus on the most critical educational outcomes that need to be measured when students are on placement within these settings [12]. Therefore, further research is needed to establish a consensus on how to evaluate student-led health care services as clinical placement providers.

Clinical placement evaluation has typically been specific to a single element of student experience, for example student confidence [13] or student satisfaction [14], or alternatively, a specific learning outcome related to a single health profession, such as clinical competency of physiotherapy students [15]. To effectively evaluate the performance of student-led health care services as providers of clinical placement, all elements of the experience that are deemed to be important must be considered together. Furthermore, an evaluation framework that can be applied to student-led health care services, which includes elements of placement performance specific to the needs and expectations of the student, the placement supervisor, the placement coordinator and to the profession is required.

Applying an evaluation framework across various health professions and health care services is not without its challenges. Previous national stakeholder consultation on use of a universal framework indicated that disciplinary and contextual differences in supervision would impact on the effectiveness of such a framework [16]. Therefore, any framework used to evaluate the quality of clinical placement needs to be consultative, collaborative, and comprehensive, so that it can accurately assess clinical placement performance across different health care settings. In addition, the framework needs to be all-encompassing and flexible so that it can be applied across different clinical placement models and different health professions [16]. A framework that can be used to effectively evaluate all clinical placement will offer the opportunity for standardization and comparability, but more importantly measure the quality of placement experience, enable quality improvement, and enhance educational opportunities [17,18].

Previous studies that have used a Delphi approach to evaluate clinical placement, or to identify factors influencing clinical placement quality, predominately exist within the medical, nursing, and allied health literature. For example, the Delphi approach has been used to engage clinical placement key stakeholders to revise clinical placement evaluation tools in physiotherapy [19], as well as to generate expert consensus between nurse clinical educators to identify important factors influencing student learning during clinical placement [20]. Additionally, a modified Delphi approach was adopted to identify key features within a quality measures framework to guide clinical placement in allied health, dentistry, medicine, and pharmacy [18]. These studies support previous notions that current tools and approaches to evaluating clinical placement quality and performance are limited in their generalizability and that key factors influencing quality in clinical placement need to be considered, that is, student learning outcomes, experience of placement, and cost of placement.

The aim of this paper, therefore, is to outline a protocol for a modified Delphi study designed to gain consensus on what is important to measure when evaluating an allied health clinical placement. This study’s objective is to gather information on areas of measurement related to student learning outcomes, experience of placement and cost of placement and then reach consensus on which of those areas need to be included in a framework for evaluation of clinical placement performance within the context of student-led health care services.

## Methods

### The Delphi Approach

A 3-round modified Delphi study design will be conducted to establish consensus on what is important to measure when evaluating clinical placement, from the perspective of students, placement supervisors, and coordinators, as experts with firsthand experiences of clinical placement.

The Delphi is a mixed methods approach to systematically collect judgments and opinions on a topic, through sequential questionnaires to gain respondents feedback on responses from earlier rounds of questioning [21]. The primary aim of the Delphi technique is to obtain consensus among a selected panel of experts. The Delphi approach will allow for drilling down into common areas of clinical placement through multiple rounds of questioning, as well as analysis of conflicting and common viewpoints through statistical methods [22]. The Delphi technique has been used extensively in higher education, health, and social sciences to inform the development of criteria, forecasting, issue prioritization, policy formation, and framework development [23,24].

Approaches to using the Delphi technique have evolved over time, which has led to some criticism on the validity of this approach. At the heart of the Delphi technique is the ability to form consensus between experts within a particular field. However, there is currently no set level for consensus [24] with consensus previously set anywhere from 51% to 100% [25]. Additionally, previous studies indicate that panel size and the area of investigation influences consensus [26] and consideration needs to be given to achieving an expert panel of an appropriate size and make up [27].

Anonymity, iteration, controlled feedback, and statistical stability of consensus are essential components of the Delphi approach [28]. However, the time it takes to complete these types of studies is often underpredicted [24] and has been found to influence low response rates in later rounds [24,25,27]. The several adaptations of the Delphi technique have also received criticism, as many Delphi studies lack detailed methodologies, influencing their reproducibility [24,28]. Additionally, the relevance of using experts to inform research has been questioned [25-27], as expert opinion is considered inferior to more highly regarded evidence-based methods [28,29]. The term *expert* has also broadened over time and is now not only used to refer to an individual’s scientific or professional expertise but can include patients or users of an intervention to create diversity within the expert panel [30]. It is not clear what the effects of a heterogeneous expert panel has on consensus building [30], however, this has become a defining feature within the Delphi technique [31].

Currently, no proven quality indicators for Delphi studies exist [30]. However, the Delphi technique does provide an opportunity to draw on the experiences and expertise of a range of key stakeholders with a diverse set of perspectives in an iterative process where complex ideas can be examined and built on to form consensus. Therefore, considering the limitations of this approach, it is crucial to plan and set standards before commencing the process to improve the robustness of the Delphi technique [28].

### Expert Panel Members

#### Overview

A minimum of 18 and maximum of 30 experts will be recruited to the expert panel, with a minimum of 6 members (hereafter referred to as *experts*) for each identified group (ie, recent allied health graduates, placement supervisors, and placement coordinators). This panel size is viewed as manageable, where available resources can be applied to vigorously promote high response rates within rounds [32]. To achieve heterogeneity within the panel, purposive sampling to ensure maximum variation in expert characteristics will be undertaken. Experts will consist of those with firsthand experience either coordinating placement, supervising placement, or undertaking clinical placement. All experts will be aged 18 years or older, be an Australian resident, and be English speaking. Additionally, clinical placement coordinators will be required to be employed in an Australian university allied health program, with involvement in coordinating or undertaking assessment of clinical placement within the past 2 years; clinical placement supervisors will be required to be located within Australia, and have had supervised allied health students from Australia-based universities within the past 2 years; and graduates will be required to have completed their allied health program within the past 12 months from an Australian university, successfully completing a clinical placement subject of an applied or immersive nature (eg, not solely an observational placement).

Consistent with the Delphi technique, the aforementioned approach to sampling will elicit expert opinion on the topic in question, and the disciplinary areas of expertise required [33]. Suitability of panel membership has been generally based on a background or experience concerning the target issue and an ability to contribute helpful inputs to the questions being asked [33]. The heterogeneity of the expert panel, as a critical feature within the Delphi technique [33], is required to maintain quality in research design and uncover the entire spectrum of opinion on the topic [27].

#### Expert Panel Member Recruitment

Potential experts will be identified, and if interested, will be invited to register through an electronic short survey sent through email. In the survey, they will be asked to confirm their interest in taking part in the study or to forward it to other potential experts meeting the target group criteria. The eligibility of potential experts who have registered will be assessed and if deemed to meet the target group criteria, will be invited to participate in the study.

Placement supervisors will be identified based on their previous or current involvement in hosting allied health students on clinical placement. Placement supervisor information from across Australia will be identified through the James Cook University placement software database (InPlace) and by snowball sampling. InPlace is a web-based software application developed by QuantumIT Pty Ltd to enable universities to identify supervisors for a given site, coordinate and manage clinical placements, and store data on placement contact information.

Placement coordinators will be identified by their previous or current involvement in coordinating clinical placement within Australian universities. Placement coordinator information will be identified through academic role descriptions on Australian university websites and membership details within discipline specific clinical placement committees.

Recent graduates will be identified by their past enrollment in a placement subject within an Australian university allied health program. This information will be identified through James Cook University alumni networks and snowball sampling whereby contact will be made with key people within clinical placement partnerships and those who hold key positions within Australian universities to identify members of the target groups who meet the inclusion criteria. Key people within clinical partnerships will include national clinical placement committed groups within professional registration bodies (eg, Exercise and Sport Science Australia, Australian Health Practitioner Regulation Agency, Occupational Therapy Australia, and Speech Pathology Australia) and existing placement arrangements with external clinical placement sites.

#### Panel Characteristics

Purposive sampling of experts to ensure equal representation across placement coordinators, placement supervisors, and recent graduates and to achieve maximum variation in expert characteristics, will be conducted using a matrix method to gain representation based on geographical location, experience, and exposure to clinical placement, as well as the allied health profession they represent. Textbox 1 outlines the expert panel selection criteria and the specific factors of variation intended to be achieved through purposive sampling techniques.

**Selection criteria and considerations for variation in expert panel members.**

**Placement coordinators:**

- Duration in the role of placement coordinator
- Geographical locations of placements coordinated, that is, rural or remote, regional, or metropolitan areas
- Types of clinical placement coordinated, for example, student-led, role-emerging, traditional, and interdisciplinary
- Allied health discipline for which they coordinate placement

**Placement supervisors:**

- Level of experience supervising students on clinical placement
- Geographical locations of previous and current employment, that is, rural or remote, regional, or metropolitan areas
- Types of clinical placement supervised, for example, student-led, role-emerging, traditional, and interdisciplinary
- Allied health discipline for which they currently supervise

**Recent graduates:**

- Level of exposure to areas of practice while on placement
- Geographical locations of placements completed, that is, rural or remote, regional, or metropolitan areas
- Types of clinical placement completed, for example, student-led, role-emerging, traditional, and interdisciplinary
- Allied health discipline from which they graduated

### Survey Design and Facilitation Within Delphi Rounds

The Delphi technique uses several rounds, whereby a series of questionnaires are provided to experts until consensus is reached [27]. Following each round, a summary of results from the previous round is provided to the experts to either gain consensus or elicit further discussion [27]. In this study, 3 or more consensus rounds will be conducted with survey questionnaires sent to each expert electronically.

Like that of a classical Delphi, the survey design will use open-ended questions, aimed at facilitating idea generation and eliciting opinion [27]. Three overarching themes, predetermined through a review of the literature, will be used to identify common areas of measurement when evaluating student-led health care services and clinical placement. These overarching themes include “learning outcomes,” “student experience,” and “cost of placement.”

A pilot questionnaire for round 1 will be tested with a small sample of people including 1 allied health placement supervisor, 1 recent graduate, and 1 allied health academic to ensure clarity of each question [34]. Textbox 2 outlines open-ended questions 1 to 5 that will be included in the round 1 questionnaire.

**Proposed round 1 survey questions.**

Question 1: Reflecting on previous experience, what is important to measure when evaluating clinical placement?

Question 2: When evaluating learning outcomes of clinical placement, please describe what variables to measure. (Provide an example where necessary)

Question 3: When evaluating student experiences of clinical placement, please describe what variables are essential to measure. (Provide an example where necessary)

Question 4: When evaluating costs associated with clinical placement, please describe what variables are essential to measure. (Provide an example where necessary)

Question 5: Is there any additional information that you would like to provide in relation to what is important to measure when evaluating clinical placement?

Question 1 is designed to set the scene and encourage the experts to respond to the question more broadly, without leading their responses. Questions 2-4 are designed to solicit information around specific areas of clinical placement related to the predetermined themes and to direct experts to consider what specific areas of clinical placement, that is, subthemes of measurement, exist when evaluating clinical placement. The final question in the survey will allow experts to discuss other areas of clinical placement evaluation, not yet covered.

Round 2 will include a structured questionnaire that will focus on areas of measurement identified in round 1 and relate to the overarching themes of learning outcomes, student experience, and cost of placement. Experts will rank the level of importance for each area of measurement using a 4-point Likert-type scale ranging from 1=not very important to 4=very important [34]. Experts will be given the opportunity to provide suggestions on any current methods of measurement for each of the proposed areas of measurement, which then will be analyzed and presented in subsequent rounds [35].

In round 3, a list of prioritized areas of measurement from the previous round will be presented. Experts will be asked to rank their level of agreement on the areas of measurement required, using 2 options: agree or disagree. Additionally, any specific methods of measurement identified in round 2 will be ranked using a 5-point Likert-type scale ranging from 1=least effective to 5=most effective [34]. Once again, experts will be invited to provide free-text comments to further explain their position.

### Data Collection

All responses will be captured using the Qualtrics web-based survey platform (version 2009; Qualtrics). For each round, each expert will be sent a unique web link to access the surveys. Expert panel members will be provided with a minimum of 4 weeks to complete all surveys within each round, with only those who participated in previous rounds being invited to participate in subsequent rounds. Weekly reminder emails will be sent to each expert until each round is completed. Once the final deadline is reached, surveys will be closed to all experts and responses will be analyzed before commencing the next round. Each survey will take no more than 40 minutes to complete and all responses will be deidentified and coded to maintain anonymity between experts.

### Data Analysis

Round 1 responses will be analyzed using thematic analysis to generate themes related to “learning outcomes,” “experience of placement,” and “cost of placement,” as well as any additional themes identified through the process. The thematic analysis will follow Braun and Clark’s [36] 6-phase framework for analyzing qualitative data [36]. These steps include becoming familiar with the data, generating initial codes, searching for themes, reviewing, and defining themes, as well as the final write-up [37]. Qualitative data analysis software, NVivo (version 12; NVivo Inc), will be used to code and group data. Additionally, triangulation will occur between the main research team (LS, FB, and RB) and an independent researcher with experience in thematic analysis to ensure validity of our assumptions through the convergence of themes from different sources [38].

Round 2 and 3 quantitative data will be analyzed using percentage of agreement to confirm whether consensus had been achieved. As identified in other Delphi protocols within health and education fields, combined scores of importance and agreement must equal ≥75% to achieve consensus [39]. In accordance with round 1, qualitative data from rounds 2 and 3 will be analyzed thematically, where common themes will be identified and presented as statements in subsequent rounds.

### Ethical Considerations

Informed consent will be obtained before participating in the research project through a unique electronic link, which will form the first page of the questionnaire in round 1. Expert anonymity will be maintained through coding of participant responses and any identifying information will not be linked with any research materials or questionnaires developed through the modified Delphi process or in the outputs that result from this research. Additionally, no compensation will be offered to selected experts for their participation in this study.

Ethical approval has been obtained through the James Cook Human Research Ethics Committee (H7541).

## Results

Data analysis is underway, with the results of the modified Delphi study expected to be submitted for publication in 2024.

## Discussion

### Overview

The aim of this modified Delphi study is to obtain consensus from a panel of experts on how to measure the salient areas of student learning outcomes, student experience, and cost of clinical placement, and to gain valuable insight into the important areas of clinical placement measurement. The outcomes of this project are intended to inform the development of a framework for evaluation of clinical placement performance.

The strength of using a modified Delphi study design for this proposed study is that it will allow for multiple perspectives from the range of stakeholders whose experiences are influenced by clinical placement performance. As a result, experts will be drawn from a variety of backgrounds to produce heterogeneity and will ensure investigation of the full scope of opinions associated with the topic [27]. The Delphi principle of anonymity also provides strength to this study’s design, as it offers an equal chance for each expert to respond in a fashion that is unbiased by the identities of the other panel members [40]. By maintaining communication through electronic means and providing deidentified results in each subsequent round, this study will ensure each expert has the opportunity to react independently, eliminating subject bias and avoiding the potential of power influenced dynamics between experts [41].

Within the Delphi approach, the notion exists that members of the panel are experts in the area of interest, where the term *expert* has been defined as “an informed individual,” a specialist in their field, or someone who is knowledgeable in a specific subject [27]. Within this study, the definition of *expert* is based on key stakeholders of clinical placement, which is commonly identified as students, universities, placement supervisors, and the organizations in which placement takes place [4,42], all of which, significantly influence clinical placement quality and performance. Incorporating the knowledge, views, and experiences of key stakeholders is critical to determining a best practice approach to the evaluation of clinical placement performance. Criticism of the expert opinion sought within the Delphi approach applies to the terminology used, determining who is an expert and what degree of expertise is required to offer opinion [27]. Expert opinion has also been criticized as a poor basis for making judgments on a topic and that researchers should consider whether the research question could be better answered through a systematic review [31,43]. However, current systematic reviews on clinical placement performance in student-led health care services identified limitations in the overall quality and generalizability of the research [10-12]. Therefore, this study will inform the critical appraisal of studies that intend to evaluate student-led health care services as providers of clinical placement and whether this is aligned to key stakeholder perspectives.

Consensus will be determined in this study using percentage of agreement, where scores of importance, effectiveness, and agreement must equal ≥75% to be deemed consensus. As seen in similarly designed studies [32,39], this approach favors the majority, but also allows for the anticipated variability in opinion from a heterogeneous expert panel. As a group of experts are unlikely to demonstrate 100% agreement, consensus forming is the essence of the Delphi technique, wherein an appropriate level of consensus needs to be determined to avoid bias [31,43]. Determining consensus remains a contentious issue with critics of the Delphi approach [27,32], however most Delphi researchers agree that the approach needs to be determined in advance and that resistance to consensus needs to be considered carefully, as this will yield new perspectives on the topic in question and will require further investigation [32,44].

### Conclusion

This protocol paper describes a modified Delphi study design, to explore key stakeholder perspectives on important areas of clinical placement performance measurement, related to learning outcomes, student experience, and cost of placement. Despite the limitations of the Delphi approach, existing research, and its ability to gain consensus through multiround expert engagement supports this methodology as appropriate to inform the development of a framework for the evaluation of clinical placement performance. As such, the outcomes of this study are intended to form part of a framework for the evaluation of allied health student-led health care services.

## Data Availability

The data sets generated and analyzed during this study are available from the corresponding author on reasonable request.
